# Cinaciguat ameliorates glomerular damage by reducing ERK1/2 activity and TGF-ß expression in type-1 diabetic rats

**DOI:** 10.1038/s41598-017-10125-3

**Published:** 2017-09-11

**Authors:** Szabina Czirok, Lilla Fang, Tamás Radovits, Gábor Szabó, Gábor Szénási, László Rosivall, Béla Merkely, Gábor Kökény

**Affiliations:** 1 Institute of Pathophysiology, Semmelweis University, Budapest, Hungary; 2 Heart and Vascular Center, Semmelweis University, Budapest, Hungary; 3 Department of Cardiac Surgery, University of Heidelberg, Heidelberg, Germany

## Abstract

Decreased soluble guanylate cyclase activity and cGMP levels in diabetic kidneys were shown to influence the progression of nephropathy. The regulatory effects of soluble guanylate cyclase activators on renal signaling pathways are still unknown, we therefore investigated the renal molecular effects of the soluble guanylate cyclase activator cinaciguat in type-1 diabetic (T1DM) rats. Male adult Sprague-Dawley rats were divided into 2 groups after induction of T1DM with 60 mg/kg streptozotocin: DM, untreated (DM, n = 8) and 2) DM + cinaciguat (10 mg/kg per os daily, DM-Cin, n = 8). Non-diabetic untreated and cinaciguat treated rats served as controls (Co (n = 10) and Co-Cin (n = 10), respectively). Rats were treated for eight weeks, when renal functional and molecular analyses were performed. Cinaciguat attenuated the diabetes induced proteinuria, glomerulosclerosis and renal collagen-IV expression accompanied by 50% reduction of TIMP-1 expression. Cinaciguat treatment restored the glomerular cGMP content and soluble guanylate cyclase expression, and ameliorated the glomerular apoptosis (TUNEL positive cell number) and podocyte injury. These effects were accompanied by significantly reduced TGF-ß overexpression and ERK1/2 phosphorylation in cinaciguat treated diabetic kidneys. We conclude that the soluble guanylate cyclase activator cinaciguat ameliorated diabetes induced glomerular damage, apoptosis, podocyte injury and TIMP-1 overexpression by suppressing TGF-ß and ERK1/2 signaling.

## Introduction

The prevalence of chronic kidney disease is estimated to be 8–16% worldwide, and was ranked 18th in the list of causes of total number of global deaths in 2010^1^. Diabetic nephropathy (DN) is the most common cause of end-stage renal disease (ESRD). Apart from cardioprotection, the renin-angiotensin-aldosterone system blockade is currently the most widely used therapy to slow the loss of renal function^2^, with additional new candidates for SGLT2 inhibition in diabetic patients^3^. However, the prevention and cure of DN is not yet solved.

DN is characterized by complex pathomechanisms including oxidative stress^4^, podocyte damage^5^ and glomerulosclerosis^6^. The accompanying accumulation of glomerular and interstitial extracellular matrix (ECM) components, such as collagen IV, is mostly driven by transforming growth factor-ß1 (TGF-β1) and connective tissue growth factor (CTGF) induced signaling pathways^7^. The accumulation of ECM components depends on the functional equilibrium of matrix metalloproteases (MMPs, which degrade the ECM), and their inhibitors (tissue inhibitors of metalloproteases (TIMPs)). The activation of TGF-ß signaling leads to MMP/TIMP imbalance, favoring fibrosis^8^.

The altered production of endothelial nitric oxide (NO) – through hemodynamic and paracrine effects between endothelial cells, podocytes and mesangial cells – and decreased glomerular cyclic 3′,5′ guanosine monophosphate (cGMP) levels are suggested to account for the expansive pathology in DN^9, 10^.

Both *in vivo* and *in vitro* studies suggest, that the NO-cGMP axis plays an important role in the maintenance of renal perfusion and glomerular filtration^11^, apart from its antifibrotic properties (due to reduced TGF-ß and extracellular matrix production^12–14^ and inhibition of cell proliferation^10^). NO binds to, and activates the soluble guanylate cyclase (sGC), which converts guanosine triphosphate (GTP) to cyclic guanosine monophosphate (cGMP), and then cGMP mediates the biological functions of NO^15^. Among hemodynamic actions^11, 12^, the NO–driven cGMP is important for normal podocyte function by maintaining the proper organization of slit diaphragm and cytoskeleton in podocytes^13, 14^.

Hyperglycaemia downregulates the NO-sGC-cGMP pathway and the reduced cGMP levels aggravate renal damage^10, 16^. Elevating cGMP levels by selective inhibition of the cGMP degrading phosphodiesterase-5 (PDE-5) has been shown to ameliorate renal disease in several animal models^17–19^, including both type 1 and type 2 diabetes^20–22^. Furthermore, diabetes leads to oxidative stress that can inactivate sGC through its heme prosthetic group, which further decreases cGMP bioavailability. The sGC activators bind to the oxidized, thus heme-free and inactive enzyme to increase enzyme activity, practically restoring the normal cGMP production in diseased (oxidized) state^23^. Cinaciguat (BAY 58-2667) is a potent sGC activator with a profile similar to organic nitrates/NO donors^24^. Cinaciguat has been shown to reduce renal fibrosis in both 5/6 nephrectomized^25^ and Dahl salt-sensitive rats^26^. However, these studies did not elucidate the molecular effects of cinaciguat on renal diseases.

Therefore, we aimed to investigate the renal molecular actions of the sGC activator cinaciguat in streptozotocin-induced type-1 diabetic rats, focusing on glomerular and podocyte damage, glomerular cGMP levels, apoptosis, cell proliferation, profibrotic signaling (TGF-ß1 CTGF, ERK1/2) and MMP/TIMP imbalance.

## Results

### Cinaciguat did not affect metabolic changes, renal hypertrophy or blood pressure

At the end of the study, diabetic rats had significantly elevated blood glucose levels and glucosuria, regardless of the treatment. The daily water intake rose markedly in all diabetic rats, but the cinaciguat-treated animals drank less than the non-treated rats (Table 1). Body weight decreased in all diabetic rats, and significant renal hypertrophy developed in both the DM and DM-Cin groups as shown by increased kidney weight/body weight ratio (Table 1). Compared to non-diabetic control groups, both DM and DM-Cin rats had lower mean arterial blood pressure (Table 1).

**Table 1:** Blood and urinary glucose levels, daily water intake, body weights and relative kidney weights of the study groups.

Parameter	Co	Co-Cin	DM	DM-Cin	P_diabetes_	P_treatment_	P_interaction_
Blood glucose (mmol l^−1^)	5.7 ± 0.4	6.1 ± 0.2	30.2 ± 1.6^a,b^	30.3 ± 3.5^a,b^	<0.001	0.698	0.748
Daily water intake (ml day^−1^)	33.4 ± 2.4	37.9 ± 2.6	239.8 ± 14.2^a,b^	154.9 ± 15.7^a,b,c^	<0.001	<0.001	<0.001
Body weight (g)	480 ± 60	478 ± 72	280 ± 27^a,b^	254 ± 46^a,b^	<0.001	0.530	0.351
Kidney weight/body weight (mg g^−1^)	2.5 ± 0.4	2.6 ± 0.3	5.6 ± 0.4^a,b^	5.9 ± 0.6^a,b^	<0.001	0.265	0.305
Mean arterial blood pressure (mmHg)	79.6 ± 6.8	80.1 ± 12.1	63.7 ± 8.5^a,b^	64.4 ± 9.8^a,b^	<0.001	0.845	0.969

Values are presented as mean ± SD (n = 10–12/group). ^a^p < 0.05 vs. Co; ^b^p < 0.05 vs. Co-Cin; ^c^p < 0.05 vs. DM (two-way ANOVA with Sidak’s multiple comparisons test).

### Cinaciguat increased serum cGMP level, urinary cGMP excretion and glomerular cGMP content in diabetic rats

**Figure 1:**
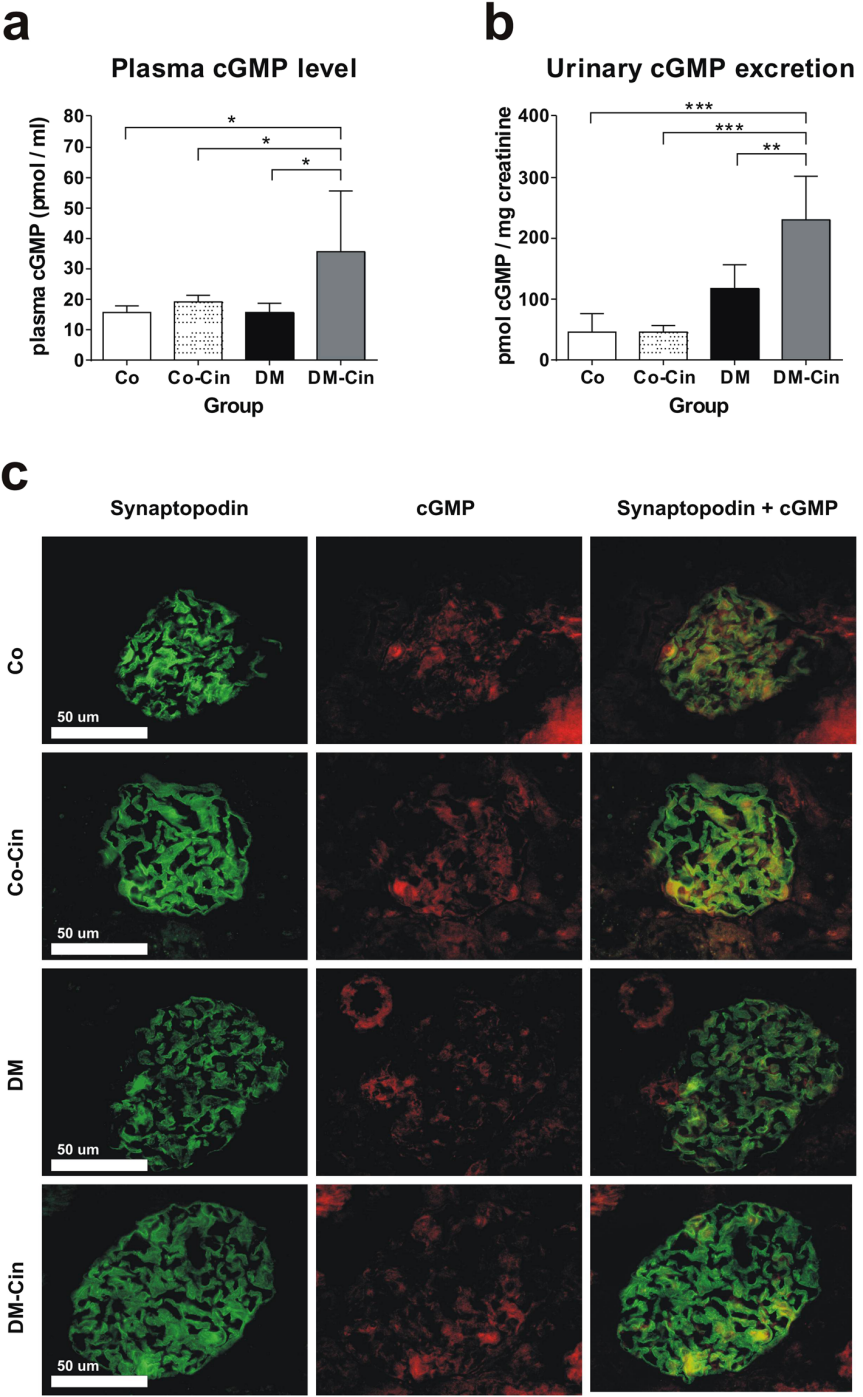
Effect of diabetes and cinaciguat treatment on cyclic guanosine monophosphate (cGMP) levels in the plasma and urine, and glomerular cGMP content. (**a**) Plasma cGMP levels of DM rats were similar to Co and Co-Cin controls, but DM-Cin rats had significantly higher circulating cGMP levels. (**b**) According to plasma cGMP, urinary cGMP excretion increased significantly in DM-Cin rats, as compared to DM or to both controls. (**c**) Double immunostaining for synaptopodin (green) and cGMP (red) depicted visible glomerular cGMP content in both Co and Co-Cin kidneys, mainly co-localized with synaptopodin (yellow). Immunoreactivity for cGMP was practically absent in glomeruli of DM rats, but it was restored in DM-Cin kidneys. Data are presented as mean ± SD (n = 8–10/group). *p < 0.05, **p < 0.01, ***p < 0.001 (two-way ANOVA with Sidak’s multiple comparison test).

Effectiveness of cinaciguat treatment was evaluated by serum cGMP levels at harvest. In non-diabetic control animals, cinaciguat did not alter plasma or urine cGMP levels. In contrast, both plasma cGMP level and urinary cGMP excretion was significantly elevated in DM-Cin rats (Fig. 1a,b). Double immunofluorescence depicted glomerular co-localization of synaptopodin and cGMP (Fig. 1c). Similar to urine and plasma cGMP levels, both control groups had similar glomerular cGMP content. However, glomerular cGMP content was reduced in DM kidneys, in contrast to urine and plasma levels. Cinaciguat preserved glomerular cGMP content, in parallel with the increased plasma and urine cGMP levels (Fig. 1c).

### Cinaciguat reduced diabetic glomerulosclerosis, proteinuria and TGF-ß overexpression

**Figure 2:**
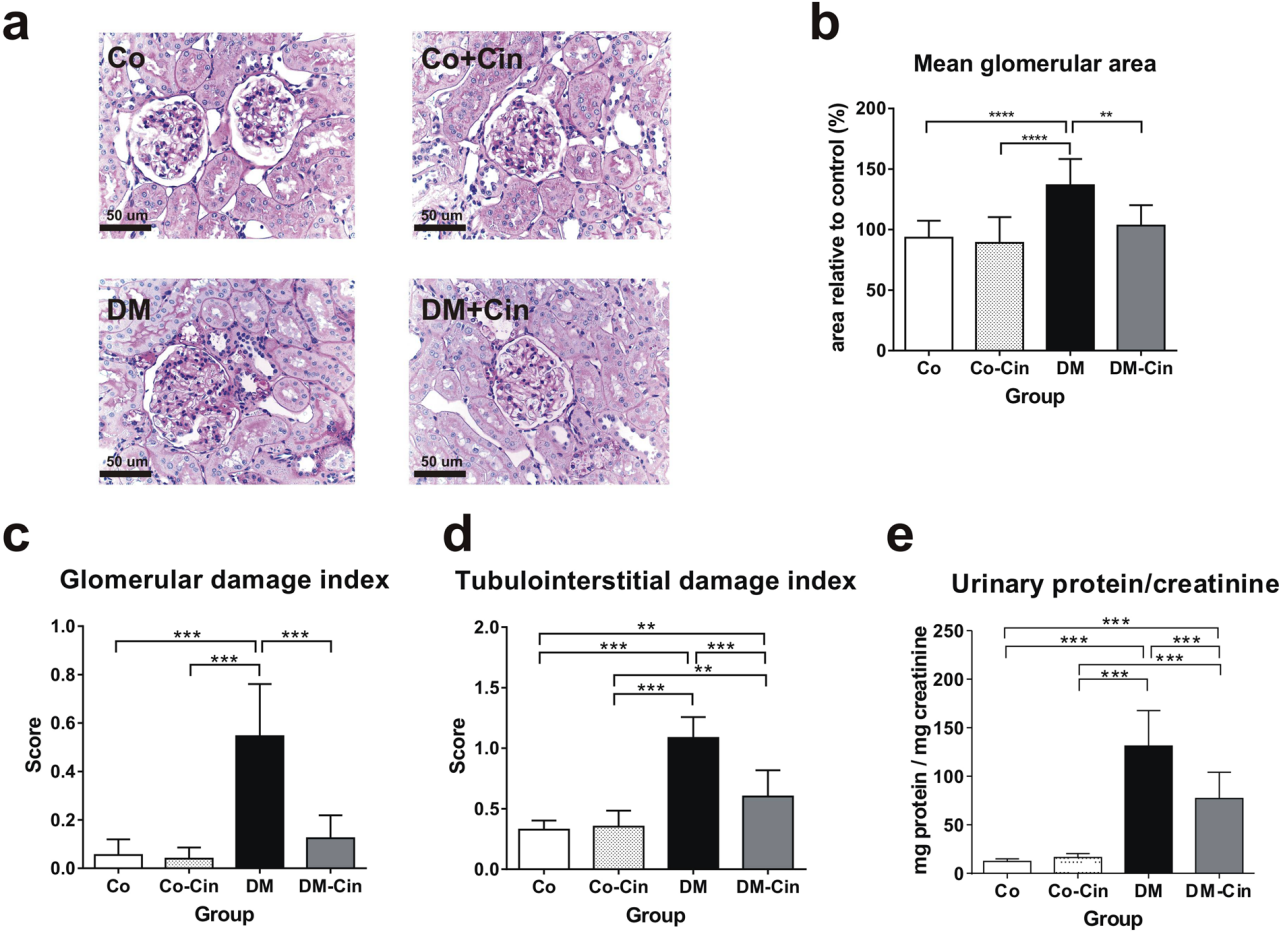
Effect of cinaciguat on kidney histology and proteinuria in diabetic rats. (**a**,**b**) Representative photomicrographs of PAS stained kidneys (400x magnification) show normal glomerular structure in both Co and Co-Cin controls, mesangial expansion and glomerular hypertrophy in DM and almost normal structure in DM-Cin groups (scale bar represents 50 μm). (**c**,**d**) Glomerular and tubulointerstitial damage index scores of each group are shown. (**e**) The extent of proteinuria was determined by urinary protein/creatinine ratio (mg protein/mg creatinine), showing significant proteinuria in DM that was reduced in DM-Cin rats. Data are presented as mean ± SD (n = 8–10/group). **p < 0.01, ***p < 0.001 (two-way ANOVA with Sidak’s multiple comparison test).

Compared to the control groups, the kidneys of untreated diabetic rats showed glomerular hypertrophy, mild mesangial expansion and tuft adhesions to Bowman’s capsule, the typical glomerular alterations in diabetes (Fig. 2a–c). Additionally, tubular dilatation and atrophy were the most prominent tubulointerstitial changes observed in untreated DM kidneys. These alterations were accompanied by marked proteinuria, as shown by the increased urinary protein/creatinine ratio of diabetic rats (Fig. 2d,e). However, cinaciguat treatment of diabetic animals significantly reduced both glomerular and tubulointerstitial changes, as well as the extent of proteinuria (Fig. 2a–e).

**Figure 3:**
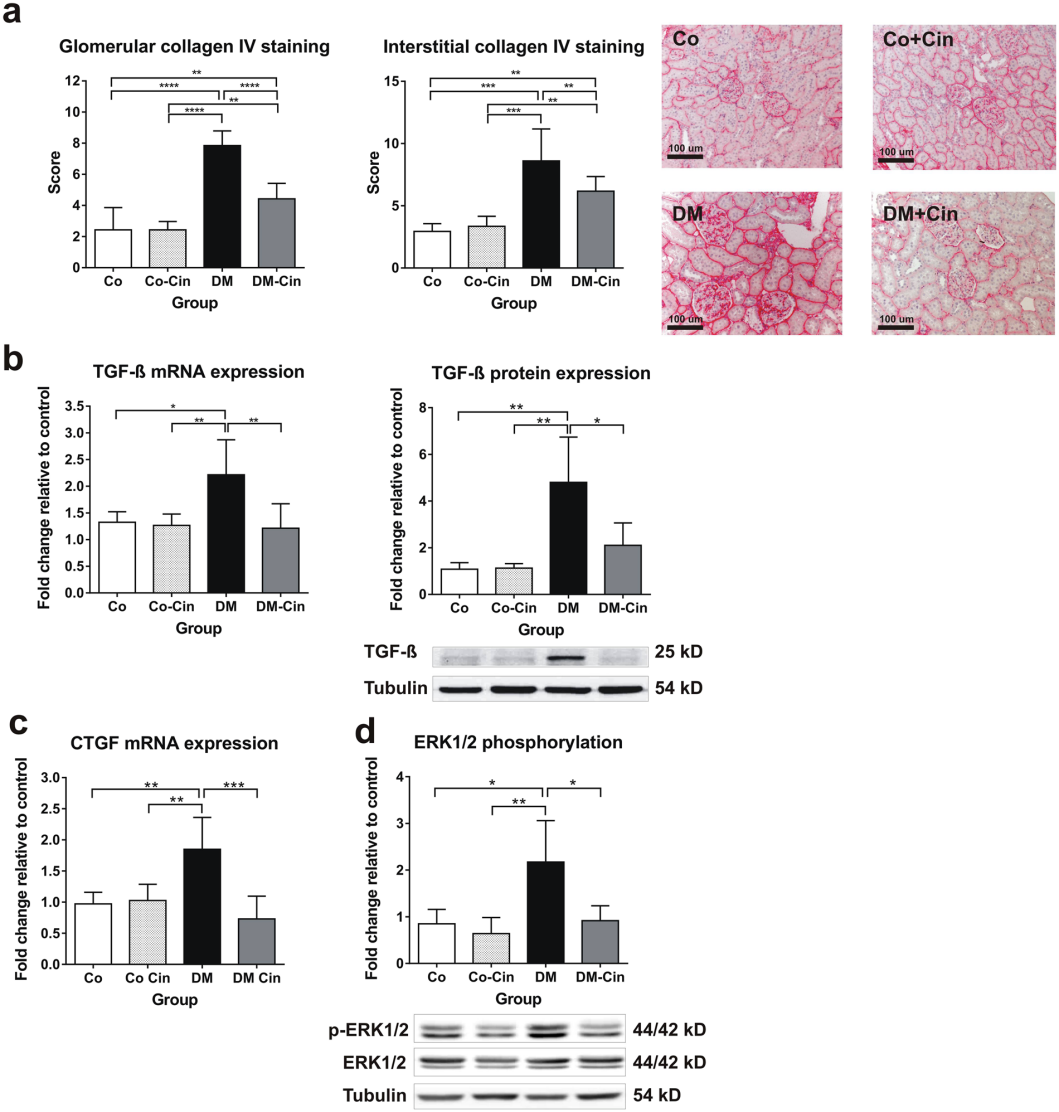
Effect of diabetes and cinaciguat on renal fibrosis. (**a**) Immunostaining for collagen IV was performed on paraffin embedded kidney sections. Representative photomicrographs (200x magnification) show moderate tubulointerstitial and glomerular staining in controls, strong tubulointerstitial and thickened glomerular expression in DM but only mild collagen IV expression in DM-Cin groups (scale bar represents 50 μm). (**b**,**c**) Compared to controls, the renal mRNA and protein expression analysis of profibrotic TGF-ß as well as CTGF mRNA expression showed a marked overexpression in DM kidneys, which was significantly attenuated in DM-Cin group. TGF-ß protein expression of each sample was normalized for tubulin expression and given as fold change relative to a calibrator. The mRNA expression were normalized for GAPDH expression using the formula *2* ^*−ΔΔCt*^. (**d**) DM kidneys showed significantly increased phosphorylation of the p44/p42 MAPK (ERK1/2) as compared to non-diabetic controls, which was inhibited by cinaciguat treatment, as seen in DM-Cin kidneys. Representative immunoblots are shown. Phospho-ERK1/2 expression was normalized for total ERK1/2 expression of each sample and given as fold change. Data are presented as mean ± SD (n = 8–10/group). **p < 0.01, ***p < 0.001 (two-way ANOVA with Sidak’s multiple comparison test).

Collagen IV immunoreactivity, as a marker of fibrosis, was markedly augmented in DM rats, but cinaciguat treated rats demonstrated significantly less collagen IV staining in both glomeruli and the interstitium (Fig. 3a).

The mRNA and protein expression of the profibrotic TGF-β_1_ was significantly elevated in the kidneys of untreated DM rats as compared to controls. Cinaciguat had no effect on TGF-β_1_ mRNA and protein expression in controls but it reduced TGF-β_1_ expression by at least 50% in diabetic rats (Fig. 3b). Accordingly, we observed reduced glomerular TGF-ß1 immunostaining in DM-Cin rats (Supplementary Figure 1). Moreover, cinaciguat was able to restore CTGF mRNA overexpression in diabetic kidneys (Fig. 3c).

Extracellular signal regulated kinases are members of the mitogen activated protein kinase (MAPK) family, which also plays an important role in fibrosis. Kidneys of untreated diabetic rats showed a 2-fold increase in ERK(1/2) phosphorylation, which was reduced to control levels by cinaciguat treatment (Fig. 3d).

### Cinaciguat attenuated diabetes-related kidney remodeling

**Figure 4:**
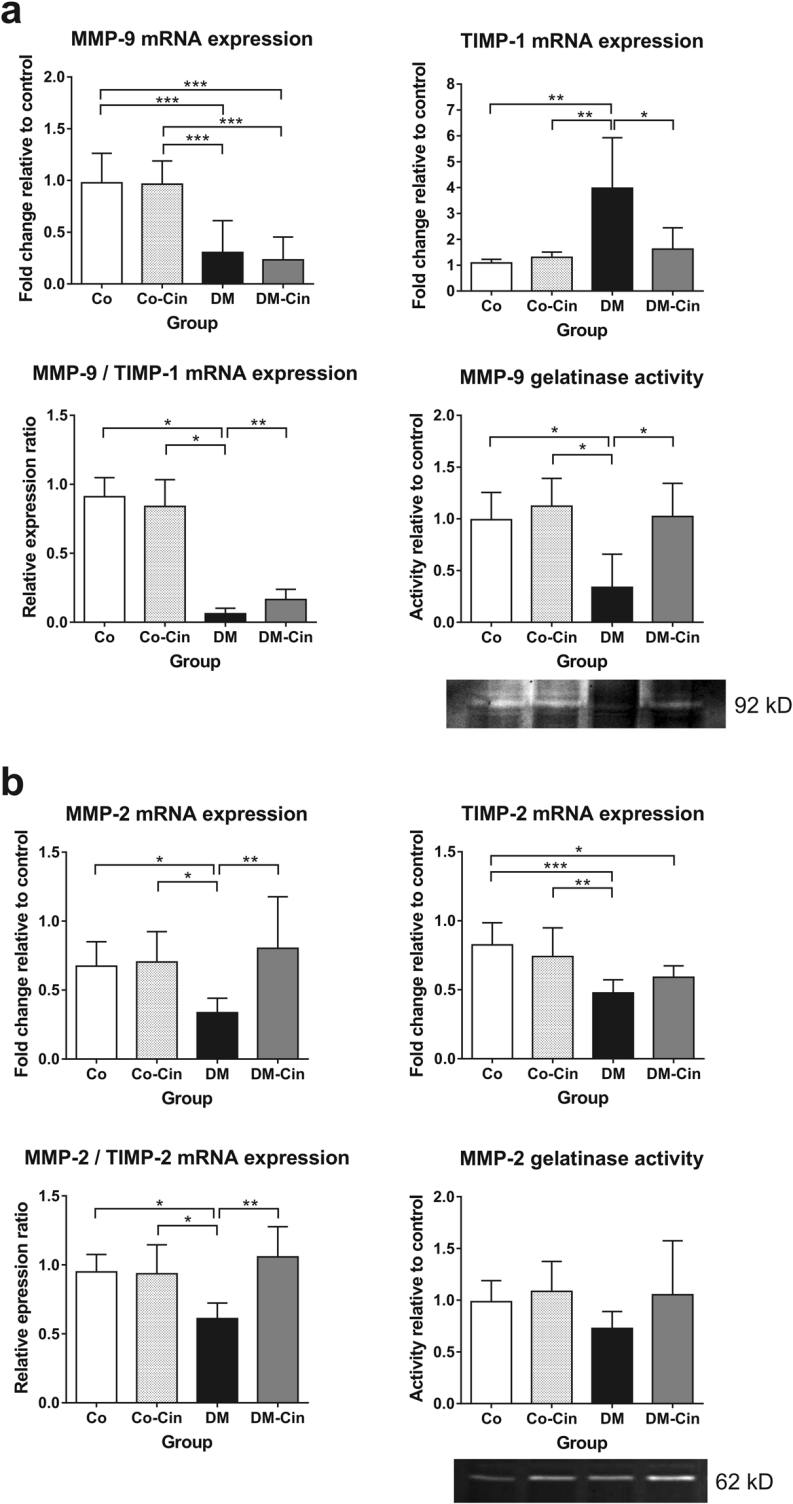
Effect of cinaciguat on the expression of renal extracellular matrix turnover components. (**a**) Compared to the non-diabetic controls, MMP-9 mRNA expression was markedly reduced in DM kidneys, accompanied by 4-fold increased TIMP-1 expression. Although cinaciguat did not alter MMP-9 expression, it attenuated TIMP-1 overexpression, which resulted in slightly better MMP-9/TIMP-1 ratio in DM-Cin kidneys, as compared to DM, supported by the MMP-9 zymography results. (**b**) The renal mRNA expression of MMP-2 dropped in DM rats, but was normalized in DM-Cin group. TIMP-2 mRNA expression reduced in both DM and DM-Cin groups, but the MMP-2/TIMP-2 imbalance was attenuated in DM-Cin kidneys. Zymography did not show, in contrast, significant changes in MMP-2 activity. Representative zymogram bands are shown. The mRNA expression was normalized for GAPDH expression using the formula *2* ^*−ΔΔCt*^. Data are presented as mean ± SD (n = 8–10/group). *p < 0.05, **p < 0.01, ***p < 0.001 (two-way ANOVA with Sidak’s multiple comparison test).

Analysis of matrix metalloproteases (MMPs) and their tissue inhibitors (TIMPs) revealed an imbalance between extracellular matrix production and degradation in diabetic kidneys, since the mRNA expression of both MMP-9 and MMP-2 were reduced (Fig. 4a,b). TIMP-1, the main inhibitor of MMP-9, showed a 4-fold overexpression in untreated diabetic kidneys as compared to controls, while renal TIMP-2 expression was repressed in both the diabetic groups. Cinaciguat was able to restore MMP-2 expression to control levels, but had no effect on MMP-9 expression. In contrast, cinaciguat ameliorated diabetic TIMP-1 overexpression without affecting TIMP-2 (Fig. 4a,b). The 92 kD gelatinase activity supported these results, showing significantly reduced 92 kD (MMP-9) activity in DM kidneys, but almost normal activity in DM-Cin kidneys. Interestingly, the 62 kD gelatinase (MMP-2) zymography did not reveal significant changes, although DM samples tended to have less MMP-2 activity.

### Cinaciguat reduced podocyte damage, cell proliferation and apoptosis in diabetic glomeruli

**Figure 5:**
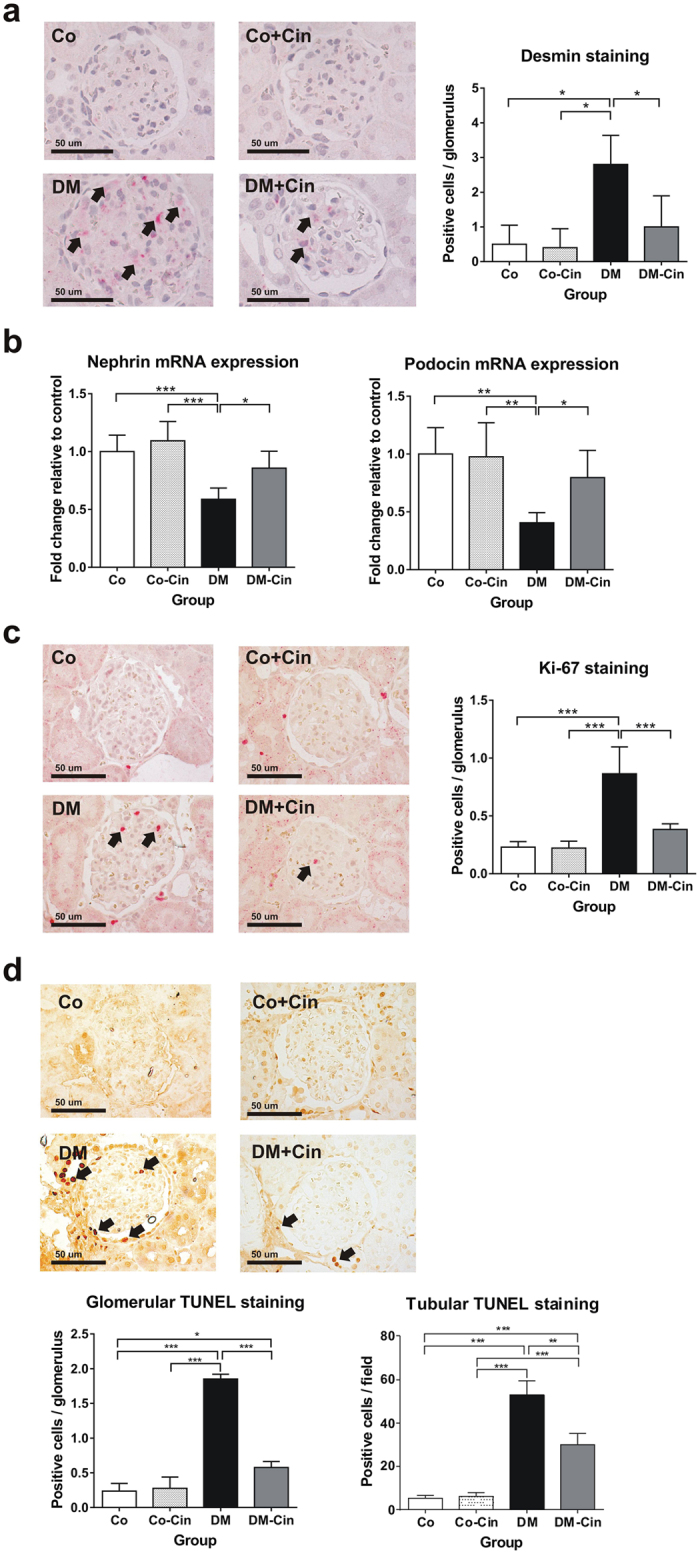
Evaluation of podocyte damage, glomerular cell proliferation and apoptosis. (**a**) Immunostaining for desmin, as marker of podocyte damage, was performed on paraffin embedded kidney sections. Representative photomicrographs (400x magnification) show no staining in non-diabetic controls, but several positive podocytes (arrows) in DM with significantly reduced staining intensity in DM-Cin kidneys (scale bar represents 50 μm). (**b**) Compared to controls, the mRNA expression of nephrin and podocin was reduced by at least 40% in DM kidneys, showing diabetic podocyte damage. Both nephrin and podocin expression was significantly ameliorated in DM-Cin kidneys. The mRNA expression was normalized for GAPDH expression using the formula *2* ^*−ΔΔCt*^. (**c**) Representative photomicrographs for Ki-67 are shown (400x magnification), as a marker of cell proliferation. We detected significantly more Ki-67 positive proliferating glomerular cells in DM rats than in non-diabetic controls, which difference was abolished by cinaciguat treatment. (**d**) TUNEL assay demonstrated a marked increase of apoptotic glomerular and tubular cell count in DM rats vs. controls, which was significantly reduced in DM-Cin kidneys. Scale bar represents 50 μm. Data are presented as mean ± SD (n = 8–10/group). *p < 0.05, **p < 0.01, ***p < 0.001 (two-way ANOVA with Sidak’s multiple comparison test).

Damaged podocytes show positive immunostaining for the intermedier filament desmin^27–29^. Strong glomerular desmin positivity was seen in the DM group as, as a marker of podocyte damage (Fig. 5a). Cinaciguat significantly attenuated desmin overexpression in podocytes of diabetic rats without any effect in controls (Fig. 5a). Nephrin and podocin mRNA expression (as further markers a podocyte injury^30, 31^) was reduced by 50% in untreated diabetic animals, but they were attenuated by cinaciguat treatment (Fig. 5b).

Kidneys of untreated diabetic rats demonstrated increased rate of glomerular cell proliferation and apoptosis, as shown by Ki-67 immunostaining and TUNEL assay, respectively. Diabetes markedly increased the number of TUNEL positive tubulus cells as well. Cinaciguat treatment reduced the rate of both glomerular cell proliferation and apoptosis, and the number of tubular apoptotic cells in diabetic animals, but had had no effect on controls (Fig. 5c,d).

### Cinaciguat attenuated the diabetes induced disruption of renal NO-sGC-cGMP-PKG signaling

**Figure 6:**
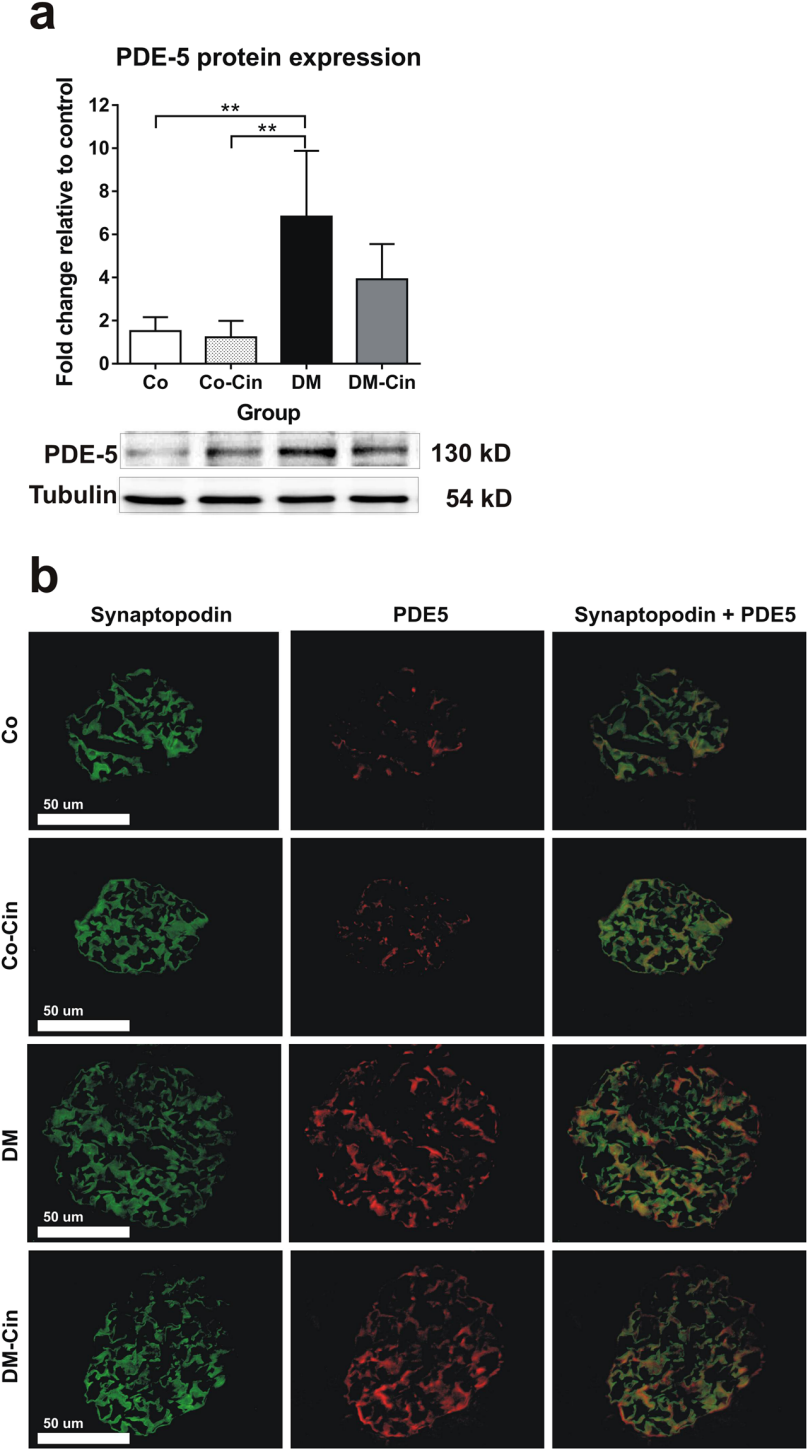
Analyses of renal PDE-5 protein expression and glomerular immunoreactivity. (**a**) As compared to non-diabetic controls, DM kidneys showed 6-fold increased PDE-5 expression. Cinaciguat treatment only tended to lower PDE-5 expression in DM-Cin kidneys. (**b**) Double immunostaining with the podocyte marker synaptopodin (green) revealed minimal basal PDE-5 expression (red) in controls but strong glomerular co-localization in DM and DM-Cin kidneys (yellow color). Bar represents 50 μm (400x magnification). Representative PDE-5 immunoblot is shown. Protein expressions were normalized to tubulin and expressed as fold change relative to a calibrator control sample. Data are presented as mean ± SD (n = 8–10/group). **p < 0.01 (two-way ANOVA with Sidak’s multiple comparison test).

**Figure 7:**
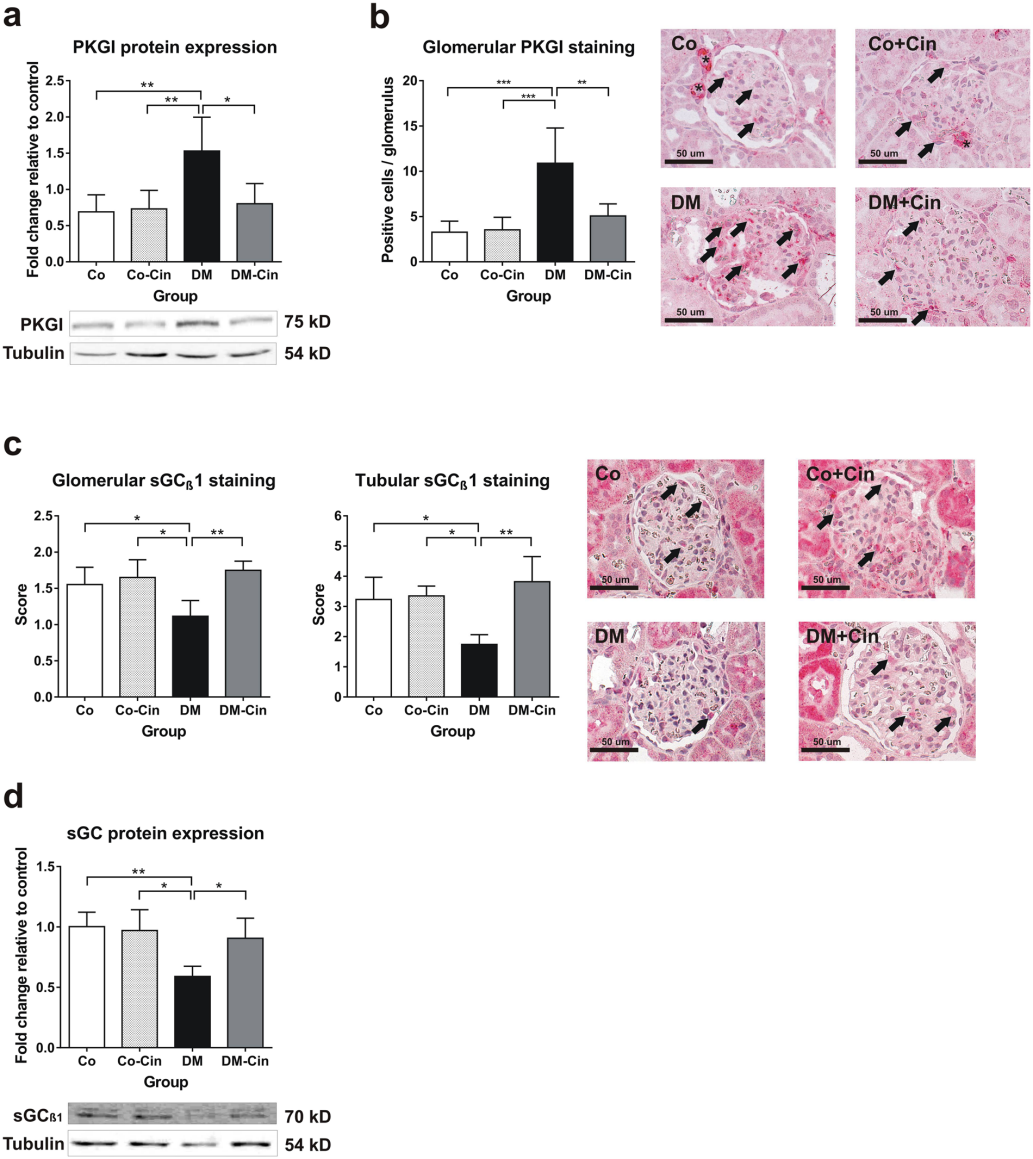
Immunoblot analysis of the renal NO-cGMP pathway components. (**a**,**b**) Glomerular and tubular guanylate cyclase (sGCβ1) immunohistochemistry depicted strong glomerular and tubular staining in non-diabetic controls (arrows), with 40% reduction of expression both in total kidney homogenates (**a**) and in glomeruli and tubuli of DM rats (**b**). Both glomerular and tubular sGC staining was significantly stronger in DM-Cin kidneys, as compared to DM. (**c**,**d**) As compared to non-diabetic controls, DM kidneys showed significant PKG overexpression **(c)** and increased number of positive glomerular cells (**d**). Cinaciguat treatment ameliorated PKG overexpression in DM-Cin as shown by immunblot (**c**) and immunostaining (**d**). Bar represents 50 μm (400x magnification). Representative immunoblots are shown. All protein expressions were normalized to tubulin and expressed as fold change relative to a calibrator control sample. Data are presented as mean ± SD (n = 8–10/group). *p < 0.05, **p < 0.01, ***p < 0.001 (two-way ANOVA with Sidak’s multiple comparison test).

Immunoblot analysis confirmed the diabetes related impairment of the NO-sGC-cGMP-PKG axis. First, the renal expression of PDE-5 was increased by 5-fold in untreated diabetic rats (Fig. 6a), and immunostaining depicted a marked glomerular PDE-5 overexpression with significant co-localization with podocyte marker synaptopodin (Fig. 6b). This was accompanied by 50% reduction in sGCß_1_ protein expression both in the glomeruli and the tubuli (Fig. 7a,b) and a significant PKG overexpression (Fig. 7c,d). Cinaciguat markedly reduced the renal PKG overexpression, and restored sGCß_1_ expression to control levels in DM-Cin rats (Fig. 7a–d).

## Discussion

The present study demonstrates that sGC activation by cinaciguat restored the glomerular cGMP content, reduced TGF-ß1 expression and ERK1/2 phosphorylation attenuating podocyte injury, proteinuria, glomerular cell proliferation and apoptosis in the rat model of type-1 diabetes. Furthermore, cinaciguat restored the renal MMP/TIMP imbalance and reduced the hyperglycemia-induced extracellular matrix accumulation.

Although many aspects of the pathomechanisms are still unclear, the NO-cGMP pathway impairment plays an important role in diabetic nephropathy. First, less NO is produced^32^, and more NO is scavenged due to increased production of superoxide. Second, the progressive inhibition and downregulation of sGC^33^ decreases cGMP production. The increased activity of the cGMP-degrading PDE-5 due to hyperglycemia further decreases the bioavailability of cGMP^21, 22^. Decreased cGMP levels results in reduced activity of the cGMP-dependent protein kinase type I (PKG), which might contribute to renal fibrosis^34, 35^. These findings were confirmed in our study by reduced glomerular cGMP content in diabetic rats, accompanied by podocyte damage and proteinuria. The NO-cGMP-sGC-PKG pathway impairment was further indicated by the glomerular PDE-5 overexpression, corroborating previous findings^21, 22^, accompanied by reduced sGC protein expression in diabetic kidneys. Despite of the increased PKG protein expression, the reduced renal cGMP bioavailability resulted in significantly increased glomerular TGF-β expression, podocyte damage (represented by elevated desmin but reduced nephrin and podocin expressions), glomerular apoptosis (as shown by TUNEL staining) and a modest increase in proliferating glomerular cell number. Despite the cGMP signaling impairment, the serum and urinary cGMP levels were not significantly reduced in DM rats as compared to controls, confirming our previous observations^22, 36^. This finding can be a consequence of diabetes-related increase in atrial natriuretic factor expression, which can activate the particulate guanylate cyclase (pGC) in other organs, resulting in a compensatory overflow of cGMP from these tissues into the plasma^37^.

In order to increase cGMP production, several sGC stimulators and sGC activators have been developed. Stimulators act synergistically with NO on the reduced sGC enzyme. In contrast, sGC activators bind only to the oxidized (therefore inactive) sGC enzyme, an advantage over stimulators in the treatment of diseases with oxidative stress^38^. Accumulating evidence suggests that both sGC stimulators^39^ and sGC activators might exert antifibrotic effects. Boustany-Kari and collegues recently reported that a sGC activator compound attenuated proteinuria and renal fibrosis on a type-2 DM rat model^40^, although the molecular background was not elucidated.

Cinaciguat preferentially activates the oxidized (inactive) enzyme^23, 24^ and has cardioprotective effects in diabetes^36^. Cinaciguat was reported to delay renal fibrosis in 5/6 nephrectomized^25^ and in Dahl salt-sensitive rats^26^. However, the regulatory effects of sGC activators on renal profibrotic signaling pathways (eg. TGF-ß1 or MAP kinases) or MMP/TIMP imbalances have not been investigated yet. Diabetes leads to glomerular and interstitial extracellular matrix (ECM) accumulation, such as collagen IV, mostly driven by TGF-β1-induced signaling pathways^7^. Hyperglycaemia induces TGF-β1 production in mesangial cells^41^. In our study, cinaciguat attenuated mesangial expansion and glomerular TGF-β_1_ expression. Apart from direct transcriptional activation of ECM proteins by TGF-β, the ECM accumulation highly depends on the balance of matrix degrading MMPs and their inhibitors (TIMPs). In our study, diabetes markedly increased TIMP-1 expression and reduced MMP-9/TIMP-1 ratio, leading to reduced MMP-9 gelatinase activity. TGF-β can induce TIMP-1 expression through both the Smad signaling^8^ and the activator protein 1 (AP-1) pathway^42^. Extracellular-regulated protein kinase 1/2 (ERK1/2) is an important player in the intracellular signal transduction system, which is involved in cell proliferation and extracellular matrix protein synthesis, partly mediated by AP-1^43^. TGF-β activates ERK1/2 in mesangial cells^44^ while ERK1/2 mediates TGF-β-induced matrix accumulation^45^ and ERK1/2 inhibition reduces TGF-β-induced collagen synthesis^46^. Hyperglycemia further promote ERK1/2 phosphorylation and subsequent upregulation of ECM components in diabetes^47^.

Both TGF-β, and ERK1/2 activation leads to increased AP-1 activity^43^. As AP-1 can induce TIMP-1^42^, the increased ERK1/2 phosphorylation and TGF-β expression explains the TIMP-1 overexpression and the progressive matrix accumulation in our untreated diabetic rats. Cinaciguat, by activating oxidized sGC and elevating cGMP levels, attenuated both renal ERK1/2 phosphorylation and TGF-β expression in diabetic kidneys. As a limitation, however, we could not investigate ERK1/2 phosphorylation of specific glomerular cells, only of the whole kidney. The recently reported sGC-TGFβ-ERK1/2 pathway crosstalk explains our observations^48^ that cinaciguat significantly reduced collagen-IV production and TIMP-1 expression.

Podocyte injury plays a major role in the pathogenesis of diabetic glomerular damage^5, 49^. High glucose increases podocyte sensitivity to ambient TGF-β1^50^ and stimulates mesangial TGF-β1 expression^5^, while TGF-β1 overproduction by mesangial cells^41, 50^ induces further podocyte damage, causing effacement^51^ and apoptosis^52^. Reduced cGMP levels might also result in hyperglycemia-induced TGF-β activation in the mesangium^53^. In our study, decreased glomerular cGMP content (including podocytes) might have contributed to cytoskeletal rearrangement in injured podocytes that caused filtration barrier leakage^13, 14^ and consequent proteinuria^54, 55^. This was evidenced by reduced expression of nephrin and podocin^5, 49^, the major components of the slit diaphragm between foot processes. In our study, DM reduced both tubular and glomerular sGC immunoreactivity, which was attenuated by cinaciguat treatment. In the glomerulus, sGC plays an important role both in mesangial cells^56^ and podocytes^57^. We suggest that the activation of oxidized sGC in podocytes and mesangial cells attenuated podocyte damage and restored nephrin and podocin expression by increasing intracellular cGMP levels in diabetic glomeruli, as shown by cGMP immunostaining. Additionally, the elevated circulating cGMP levels observed in cinaciguat treated diabetic rats might further reduce the glomerular activation of latent TGF-β1, as supported by reduced glomerular TGF-β1 immunostaining^34^.

One should also consider that elevated serum cGMP levels might result in blood pressure effects that consequently can alter renal damage. The sGC stimulator riociguat has been shown to reduce blood pressure and renal fibrosis in models of hypertension^39^. In our study, however, both diabetic groups had significantly lower MAP, which was not affected by cinaciguat. As both diabetic groups exhibited significant glucosuria, we presume that the osmotic polyuria might be responsible for the lower MAP^58^. We also have to consider the metabolic acidosis which could influence renal function, as insulin supplementation was not used in our study^59^. However, both diabetic groups had comparable weight loss and blood glucose levels, which indicates that cinaciguat had no effect on metabolic parameters. Nevertheless, we propose that the beneficial effects of cinaciguat treatment were independent of blood pressure or ketoacidosis in our study.

In terms of clinical translation, another limitation of our study is that diabetic rats did not develop hypertension or severe glomerulosclerosis. These are common clinical features of diabetic nephropathy patients and give rationale for combination treatments including RAS blockade. In a combined hypertensive-diabetic mouse model, the sGC stimulator riociguat has been shown to improve nephropathy on top of RAS blockade^60^. In the present study, our major goal was to elucidate the specific renal action of cinaciguat in monotherapy. Further animal studies are needed to investigate the possible additive therapeutic potential of sGC activator cinaciguat in combination with RAS blockade.

## Conclusions

In our study on the rat model of T1DM, the NO-independent chronic activation of sGC by cinaciguat effectively restores glomerular cGMP levels, and attenuates diabetic podocyte damage, glomerular apoptosis and fibrosis through suppression of TGF-ß overproduction and ERK1/2 phosphorylation. To the best of our knowledge, this is the first *in vivo* study showing the blood pressure independent renal molecular effects of chronic cinaciguat treatment in diabetes. Our observation and previous reports suggest the possible use of cinaciguat as a new regimen in the treatment of DN.

## Methods

### Animals

Male Sprague-Dawley rats (250–300 g, Charles River, Sulzfeld, Germany) were housed at a constant temperature of 22 ± 2 °C with 12 h light/dark cycles, had access to standard rodent chow and water ad libitum.

The investigation conforms to the *Guide for the Care and Use of Laboratory Animals* published by the US National Institutes of Health (NIH Publication No. 85–23, revised 1996). All procedures and handling of animals during the investigations were reviewed and approved by the institutional and national ethical committees for animal experimentation (permission number: 22.1/1162/3/2010).

### Induction of diabetes mellitus

Type 1 diabetes mellitus was induced with a single intraperitoneal dose of freshly dissolved streptozotocin (STZ, 60 mg/kg) in 0.1 mol/L citrate buffer. Control animals received buffer only. After 72 h, blood glucose concentration was determined using a digital blood glucose meter and test strips (Accu-Chek® Sensor, Roche Inc., Mannheim, Germany). Only animals with random blood glucose level > 15 mmol/l were considered diabetic and were included in the study.

### Experimental groups, treatment protocol

Diabetic rats were randomized to diabetic control (DM, n = 8) and cinaciguat treatment (DM-Cin, n = 8) groups. Rats injected only with citrate buffer instead of streptozotocin served as non-diabetic controls (Co, n = 10) plus there was an additional non-diabetic control group with cinaciguat treatment (Co-Cin, n = 10). The rats were treated for 8 weeks with the soluble guanylate cyclase activator, cinaciguat (Co-Cin and DM-Cin group, 10 mg kg^−1^ day^−1^ suspended in 0.5% methylcellulose solution or with vehicle (Co and DM groups) per os via oral gavage. Water bottles were filled every morning with the same amount of fresh tap water and daily water intake was measured. Body and kidney weights were measured at the time of harvest.

### Blood pressure measurements, blood and urine chemistries

At the end of the treatment period, rats were anesthetized with i.p. ketamine (100 mg/kg) and xylazine (3 mg/kg) and were placed on controlled heating pads. Arterial blood pressure was recorded by 2 F microtip pressure-volume catheter (SPR-838, Millar Instruments, Houston, TX, USA), and mean arterial pressure (MAP) was computed.

Blood samples were taken from the inferior caval vein. Urine samples were obtained by sterile puncture of the urinary bladder. Serum glucose levels as well as urine creatinine concentration were determined photometrically on a Reflotron analyzer (Roche, Boehringer-Mannheim, Mannheim, Germany). Urine protein concentration was measured using the BCA Protein Assay (Pierce Thermo Scientific, Rockford, USA), and urinary protein/creatinine ratios were calculated.

Plasma and urine levels of cyclic guanosine monophosphate (cGMP) were determined by enzyme immunoassay (EIA) using a commercial kit (Amersham cGMP EIA Biotrak System, GE Healthcare, Buckinghamshire, UK). Plasma cGMP concentration was evaluated as µmol/ml. Urine cGMP concentration (pmol/ml) was normalized for creatinine (mg/ml) and urinary cGMP excretion was calculated as pmol cGMP/mg creatinine.

### Renal histology, immunohistochemistry and TUNEL staining

Periodic-acid Schiff (PAS) staining was performed on formalin fixed, paraffin embedded kidney samples to assess glomerular and tubular damage^22^. The glomerular score of each animal was derived as the arithmetic mean of 60 glomeruli (400x magnification). The tubulointerstitial damage index (dilatation, atrophy, hyaline in tubular lumen, visible detachment of tubular cells, interstitial infiltration of mononuclear cells and interstitial fibrosis) was assessed as previously described^22^. Additionally, the average area of 30 glomeruli per kidney sections in 4 samples per group were calculated with ImageJ software^61^.

Immunohistochemistry was performed on paraffin sections, using the avidin-biotin method, as previously described^22^. Citrate buffer pH 6.0 was used for antigen retrieval, and slides were incubated with primary antibodies overnight at 4 °C (rabbit polyclonal anti-collagen IV, 1:1000, AbD Serotec, Bio-Rad, Puchheim, Germany; mouse monoclonal anti-desmin, 1:50, Dako Cytomation, Frank Diagnosztika, Budapest, Hungary; rabbit polyclonal anti-sGC_ß1_ 1:500, Novus Biologicals, Littleton, CO, USA; rabbit polyclonal anti-PKGI 1:100, Enzo, Farmingdale, NY, USA; rabbit monoclonal anti-Ki-67 1:1000, Abcam, Cambridge, UK) then with appropriate secondary antibodies (BioGenex, San Ramon, CA, USA) for 30 min and developed using Fast Red (Dako).

In order to detect DNA strand breaks as marker of apoptosis, terminal deoxynucleotidyl transferase dUTP nick end labeling (TUNEL) assay was performed using a commercial kit (DeadEnd Colorimetric TUNEL System, Promega, Mannheim, Germany) on paraffin embedded kidney sections, according to the manufacturer’s protocol.

Immunohistochemical and TUNEL assays were evaluated in a blinded manner. Reactivity of glomerular desmin (400x magnification), Ki-67 and TUNEL staining were evaluated by counting positive cells per glomerulus in each field of the section, and the average of positive cells/glomerulus per specimen was calculated. Tubular TUNEL reactivity was assessed at 200x magnification as an average of positive cells per field. Collagen IV and sGC_ß1_ staining were quantified as follows: intensity score (1 = weak staining, 2 = intermediate staining, 3 = extensive staining) and area score (1 = up to 10% positive cells, 2 = 11–50% positive cells, 3 = 51–80% positive cells, 4: > 80% positive cells) was assessed, and an average score was calculated for each field of view (intensity score multiplied by area score).

Cyclic GMP and synaptopodin, as well as PDE-5 and synaptopodin double immunostaining were performed on frozen kidney sections. Briefly, sections were fixed with 4% paraformaldehyde for cGMP or ice-cold acetone for PDE-5, then rehydrated with PBS, blocked using 5% donkey serum, and then incubated with primary antibodies (rabbit polyclonal anti-cGMP, 1:1000, Abcam, or rabbit polyclonal anti-PDE-5a, 1:200, Enzo, and mouse monoclonal anti-synaptopodin, 1:500, Fitzgerald Industries, Acton, MA, USA), followed by secondary antibodies (Dylight-488 conjugated donkey anti-mouse and Cy3 conjugated donkey anti-rabbit, both at 1:500, Jackson Immunoresearch, West Grove, PA, USA), and then washed, mounted and analyzed under fluorescent microscope.

TGF-ß and nephrin double staining was performed similarly, using mouse monoclonal anti-TGFß (1:200, R&D Systems, Minneapolis, MN, USA) with guinea pig polyclonal anti-nephrin (1:200, Fitzgerald Industries) as primary antibodies, and Cy3-comjugated donkey anti-mouse and Dylight488 conjugated anti-guinea pig (both at 1:200, Jackson Immunoresearch) as secondary antibodies.

### Immunoblot

Kidney samples (20 mg) were homogenized in RIPA lysis buffer containing complete protease inhibitor cocktail (Roche). Protein concentration was determined by the BCA Assay. Samples were mixed with 2x Laemmli buffer and boiled. Equal amounts of protein (40 µg) were separated on 10% SDS-polyacrylamide gel, transferred to nitrocellulose membranes and blocked with 5% skim milk in Tris-buffered saline (TBS), containing 0.1% Tween-20. Membranes were incubated overnight at 4 °C with primary antibodies (rabbit polyclonal anti-TGF-β_1_ antibody 1:500, SantaCruz Biotechnology, Santa Cruz, CA, USA; rabbit polyclonal anti-PKG 1:2000, Enzo, Farmingdale, NY, USA; rabbit polyclonal anti-sGC_ß1_ 1:2000, Novus Biologicals; rabbit polyclonal anti-PDE5a 1:1000, Enzo; p44/42 MAPK (ERK1/2) and phospho-p44/42 MAPK (pERK1/2) (Thr202/Tyr204) both at 1:1000, Cell Signaling, Danvers, MA, USA), then washed and incubated with peroxidase-conjugated secondary antibody (anti-mouse IgG or anti-rabbit IgG, 1:2000, Cell Signaling). Blots were visualized by ECL detection kit (Pierce Thermo). Original immunoblots are shown in Supplementary Figure 2 for PDE-5, TGF-ß1 and p-ERK and in Supplementary Figure 3 for PKG and sGC_ß1_.

### Gelatin zymography

MMP-2 and MMP-9 activity was estimated by gelatin zymography. Kidneys were homogenized in lysis buffer (50 mM TRIS-HCl pH 7.5; 500 mM NaCl; 5 mM CaCl_2_) containing EDTA-free complete protease inhibitor cocktail (Roche, Indianapolis, IN, USA) and protein concentration was determined using the BCA Protein Assay Kit (Pierce Thermo Scientific). Equal quantities (40 µg) of protein were loaded in 8% polyacrylamide gels containing 0.1% gelatin, and electrophoresis was performed under non-reducing conditions. Following electrophoresis, gels were washed with 2.5% Triton X-100 for 20 min to remove sodium dodecyl sulfate. Gels were incubated at 37 °C for 18 h (for MMP-2) or 24 h (for MMP-9) in renaturing buffer (50 mM TRIS-HCl; 5 Mm CaCl_2_; 1 µM ZnCl_2_; 0.02% NaN_3_), washed in fixing buffer (30% methanol, 10% acetic acid), and stained with 0.5% (w/v) Coomassie Blue R-250 (Biomol, Hamburg, Germany) in fixing buffer for 30 min at room temperature. Gels were destained with fixing buffer. Gelatinolytic activity was visualized as a clear band in a blue background. Band intensity was quantified using ImageJ^61^. Original zymogram is shown in Supplementary Figure 4.

### Quantitative RT-PCR

50 mg of whole kidneys were homogenized and total RNA was isolated according to the manufacturer’s protocol (TRIzol, Thermo Fisher Scientific, Waltham, MA, USA). 2 μg RNA was reverse transcribed (High Capacity cDNA Reverse Transcription kit, Thermo) using random primers. PCR reactions were performed on a BioRad CFX thermal cycler (BioRad, Hercules, CA, USA) using the Power SYBR Green PCR Master Mix (Thermo). Specificity and efficiency of the PCR reaction was confirmed with melting curve and standard curve analysis, respectively. Duplicate samples were normalized to glyceraldehyde-3-phosphate dehydrogenase (*Gapdh*) expression. Mean values are expressed with the formula 2^*−*ΔΔ*Ct*^. Primer sequences were as follows: *Ctgf* forward: ATGCTGTGAGGAGTGGGTGT; *Ctgf* reverse: GGCCAAATGTGTCTTCCAGT; *Tgfb1* forward 5-ACCATCCATGACATGAACC-3; *Tgfb1* reverse 5-TCATGTTGGACAACTGCTCC-3; *Mmp*2 forward: 5-GCTGATACTGACACTGGTACTG-3; *Mmp*2 reverse: 5-CACTGT CCGCCAAATAAACC-3; *Mmp9* forward: 5-CTTGAAGTCTCAGAAGGTGGATC-3; *Mmp9* reverse: 5-CGCCAGAAGTATTTGTCATGG-3; *Timp1* forward: 5-TTTCTG CAACTCGGACCTG-3; *Timp1* reverse: 5-ACAGCGTCGAATCCTTTGAG-3; *Timp*2 forward: 5-TCGAATTTATCTACACGGCCC-3; *Timp2* reverse: 5-GGCACAATAAAG TCACAGAGGG-3; *Timp3* forward: 5-AAGGCAAGATGTACACAGGG-3; *Timp3* reverse: 5-TGGAGGTCACAAAGCAAGG-3; *Nphs1* (*nephrin*) forward: 5-GCCTCTTGACCAT CGCTAATG-3; *Nphs1* reverse: 5-GACAACCTTCAGTCCCAG TG-3; *Nphs2* (*podocin*) forward: 5-TCCCTTTTCCATCTGGTTCTG-3; *Nphs2* reverse: 5-CTTGTGATAGGTGTCCAGGC-3; *Gapdh* forward: 5-CAATGACCCCTTCATTGA CC-3; *Gapdh* reverse: 5-CGCCAGTAGACTCCACAACA-3.

### Statistics

The statistical analyses were performed with Prism 6 (GraphPad, La Jolla, CA, USA) on a personal computer. All the data are presented as mean ± standard deviation (SD). Data were eveluated using two-factorial analysis of variance (two-way ANOVA, with ‘diabetes’ and ‘Cinaciguat treatment’ as factors) to detect independent effects of the factors (p _diabetes_, p _treatment_) and significant diabetes × treatment interactions (p _interaction_). Sidak’s multiple comparisons test was performed as *post hoc* test to evaluate differences between groups. The level of significance was set to p < 0.05.

### Ethical Approval Statement

All procedures and handling of animals during the investigations were reviewed and approved by the local Ethical Committee for Animal Experimentation at Semmelweis University and the Directorate of Food Chain Safety and Animal Health of the Pest County Government Office (permission number: 22.1/1162/3/2010). The investigation conforms to the *Guide for the Care and Use of Laboratory Animals* published by the US National Institutes of Health (NIH Publication No. 85–23, revised 1996) and the European Directive 2010/63/EU.

## Electronic supplementary material

Supplementary Figures

## References

[1] Jha V (2013). Chronic kidney disease: global dimension and perspectives. Lancet.

[2] Brewster UC, Setaro JF, Perazella MA (2003). The renin-angiotensin-aldosterone system: cardiorenal effects and implications for renal and cardiovascular disease states. The American journal of the medical sciences.

[3] Ito D (2017). Effects of Ipragliflozin on Diabetic Nephropathy and Blood Pressure in Patients With Type 2 Diabetes: An Open-Label Study. Journal of clinical medicine research.

[4] Suzuki D (1999). Immunohistochemical evidence for an increased oxidative stress and carbonyl modification of proteins in diabetic glomerular lesions. Journal of the American Society of Nephrology: JASN.

[5] Wolf G, Chen S, Ziyadeh FN (2005). From the periphery of the glomerular capillary wall toward the center of disease: podocyte injury comes of age in diabetic nephropathy. Diabetes.

[6] Mauer SM (1994). Structural-functional correlations of diabetic nephropathy. Kidney international.

[7] Ziyadeh FN (2000). Long-term prevention of renal insufficiency, excess matrix gene expression, and glomerular mesangial matrix expansion by treatment with monoclonal antitransforming growth factor-beta antibody in db/db diabetic mice. Proc Natl Acad Sci USA.

[8] Medina, C. *et al*. Transforming growth factor-beta type 1 receptor (ALK5) and Smad proteins mediate TIMP-1 and collagen synthesis in experimental intestinal fibrosis. *J Pathol* **224**, 461–472. 10.1002/path.2870 21465486

[9] Prabhakar SS (2004). Role of nitric oxide in diabetic nephropathy. Seminars in nephrology.

[10] Craven PA, Studer RK, DeRubertis FR (1994). Impaired nitric oxide-dependent cyclic guanosine monophosphate generation in glomeruli from diabetic rats. Evidence for protein kinase C-mediated suppression of the cholinergic response. The Journal of clinical investigation.

[11] Ballermann BJ, Marsden PA (1991). Endothelium-derived vasoactive mediators and renal glomerular function. Clin Invest Med.

[12] Raij L, Baylis C (1995). Glomerular actions of nitric oxide. Kidney international.

[13] Pavenstadt H (2000). Roles of the podocyte in glomerular function. Am J Physiol Renal Physiol.

[14] Sharma R, Lovell HB, Wiegmann TB, Savin VJ (1992). Vasoactive substances induce cytoskeletal changes in cultured rat glomerular epithelial cells. Journal of the American Society of Nephrology: JASN.

[15] Pacher P, Obrosova IG, Mabley JG, Szabo C (2005). Role of nitrosative stress and peroxynitrite in the pathogenesis of diabetic complications. Emerging new therapeutical strategies. Current medicinal chemistry.

[16] Prabhakar S, Starnes J, Shi S, Lonis B, Tran R (2007). Diabetic nephropathy is associated with oxidative stress and decreased renal nitric oxide production. Journal of the American Society of Nephrology: JASN.

[17] Rodriguez-Iturbe B (2005). Early treatment with cGMP phosphodiesterase inhibitor ameliorates progression of renal damage. Kidney international.

[18] Dousa TP (1998). Signaling role of PDE isozymes in pathobiology of glomerular mesangial cells. Studies *in vitro* and *in vivo*. Cell Biochem Biophys.

[19] Lledo-Garcia E (2007). Sildenafil improves immediate posttransplant parameters in warm-ischemic kidney transplants: experimental study. Transplant Proc.

[20] Jeong KH (2009). Effects of sildenafil on oxidative and inflammatory injuries of the kidney in streptozotocin-induced diabetic rats. Am J Nephrol.

[21] Kuno Y, Iyoda M, Shibata T, Hirai Y, Akizawa T (2011). Sildenafil, a phosphodiesterase type 5 inhibitor, attenuates diabetic nephropathy in non-insulin-dependent Otsuka Long-Evans Tokushima Fatty rats. British journal of pharmacology.

[22] Fang L (2013). Selective phosphodiesterase-5 (PDE-5) inhibitor vardenafil ameliorates renal damage in type 1 diabetic rats by restoring cyclic 3′,5′ guanosine monophosphate (cGMP) level in podocytes. Nephrology, dialysis, transplantation: official publication of the European Dialysis and Transplant Association - European Renal Association.

[23] Stasch JP, Pacher P, Evgenov OV (2011). Soluble guanylate cyclase as an emerging therapeutic target in cardiopulmonary disease. Circulation.

[24] Evgenov OV (2006). NO-independent stimulators and activators of soluble guanylate cyclase: discovery and therapeutic potential. Nature reviews. Drug discovery.

[25] Kalk P (2006). NO-independent activation of soluble guanylate cyclase prevents disease progression in rats with 5/6 nephrectomy. British journal of pharmacology.

[26] Hoffmann LS, Kretschmer A, Lawrenz B, Hocher B, Stasch JP (2015). Chronic Activation of Heme Free Guanylate Cyclase Leads to Renal Protection in Dahl Salt-Sensitive Rats. PloS one.

[27] Floege J (1995). Basic fibroblast growth factor augments podocyte injury and induces glomerulosclerosis in rats with experimental membranous nephropathy. The Journal of clinical investigation.

[28] Joles JA (2000). Early mechanisms of renal injury in hypercholesterolemic or hypertriglyceridemic rats. Journal of the American Society of Nephrology: JASN.

[29] Herrmann A, Tozzo E, Funk J (2012). Semi-automated quantitative image analysis of podocyte desmin immunoreactivity as a sensitive marker for acute glomerular damage in the rat puromycin aminonucleoside nephrosis (PAN) model. Experimental and toxicologic pathology: official journal of the Gesellschaft fur Toxikologische Pathologie.

[30] Alfano M (2015). Full-length soluble urokinase plasminogen activator receptor down-modulates nephrin expression in podocytes. Scientific reports.

[31] Blattner SM (2013). Divergent functions of the Rho GTPases Rac1 and Cdc42 in podocyte injury. Kidney international.

[32] Satoh M (2005). NAD(P)H oxidase and uncoupled nitric oxide synthase are major sources of glomerular superoxide in rats with experimental diabetic nephropathy. Am J Physiol Renal Physiol.

[33] Meurer S (2009). Nitric oxide-independent vasodilator rescues heme-oxidized soluble guanylate cyclase from proteasomal degradation. Circulation research.

[34] Wang S, Shiva S, Poczatek MH, Darley-Usmar V, Murphy-Ullrich JE (2002). Nitric oxide and cGMP-dependent protein kinase regulation of glucose-mediated thrombospondin 1-dependent transforming growth factor-beta activation in mesangial cells. The Journal of biological chemistry.

[35] Cui W (2014). Increasing cGMP-dependent protein kinase activity attenuates unilateral ureteral obstruction-induced renal fibrosis. Am J Physiol Renal Physiol.

[36] Matyas C (2015). The soluble guanylate cyclase activator cinaciguat prevents cardiac dysfunction in a rat model of type-1 diabetes mellitus. Cardiovascular diabetology.

[37] Hamet P, Pang SC, Tremblay J (1989). Atrial natriuretic factor-induced egression of cyclic guanosine 3′:5′-monophosphate in cultured vascular smooth muscle and endothelial cells. The Journal of biological chemistry.

[38] Stasch JP, Schlossmann J, Hocher B (2015). Renal effects of soluble guanylate cyclase stimulators and activators: a review of the preclinical evidence. Current opinion in pharmacology.

[39] Sharkovska Y (2010). Nitric oxide-independent stimulation of soluble guanylate cyclase reduces organ damage in experimental low-renin and high-renin models. Journal of hypertension.

[40] Boustany-Kari CM (2016). A Soluble Guanylate Cyclase Activator Inhibits the Progression of Diabetic Nephropathy in the ZSF1 Rat. The Journal of pharmacology and experimental therapeutics.

[41] Wolf G, Schroeder R, Zahner G, Stahl RA, Shankland SJ (2001). High glucose-induced hypertrophy of mesangial cells requires p27(Kip1), an inhibitor of cyclin-dependent kinases. The American journal of pathology.

[42] Hall MC (2003). The comparative role of activator protein 1 and Smad factors in the regulation of Timp-1 and MMP-1 gene expression by transforming growth factor-beta 1. The Journal of biological chemistry.

[43] Karin M (1995). The regulation of AP-1 activity by mitogen-activated protein kinases. The Journal of biological chemistry.

[44] Hayashida T, Poncelet AC, Hubchak SC, Schnaper HW (1999). TGF-beta1 activates MAP kinase in human mesangial cells: a possible role in collagen expression. Kidney international.

[45] Isono M, Cruz MC, Chen S, Hong SW, Ziyadeh FN (2000). Extracellular signal-regulated kinase mediates stimulation of TGF-beta1 and matrix by high glucose in mesangial cells. Journal of the American Society of Nephrology: JASN.

[46] Hayashida T, Decaestecker M, Schnaper HW (2003). Cross-talk between ERK MAP kinase and Smad signaling pathways enhances TGF-beta-dependent responses in human mesangial cells. FASEB journal: official publication of the Federation of American Societies for Experimental Biology.

[47] Dlugosz JA (2000). Stretch-induced mesangial cell ERK1/ERK2 activation is enhanced in high glucose by decreased dephosphorylation. Am J Physiol Renal Physiol.

[48] Beyer C (2015). Stimulation of the soluble guanylate cyclase (sGC) inhibits fibrosis by blocking non-canonical TGFbeta signalling. Annals of the rheumatic diseases.

[49] Jefferson JA, Shankland SJ, Pichler RH (2008). Proteinuria in diabetic kidney disease: a mechanistic viewpoint. Kidney international.

[50] Iglesias-de la Cruz MC (2002). Effects of high glucose and TGF-beta1 on the expression of collagen IV and vascular endothelial growth factor in mouse podocytes. Kidney international.

[51] Dessapt C (2009). Mechanical forces and TGFbeta1 reduce podocyte adhesion through alpha3beta1 integrin downregulation. Nephrology, dialysis, transplantation: official publication of the European Dialysis and Transplant Association - European Renal Association.

[52] Wu DT, Bitzer M, Ju W, Mundel P, Bottinger EP (2005). TGF-beta concentration specifies differential signaling profiles of growth arrest/differentiation and apoptosis in podocytes. Journal of the American Society of Nephrology: JASN.

[53] Wang S, Skorczewski J, Feng X, Mei L, Murphy-Ullrich JE (2004). Glucose up-regulates thrombospondin 1 gene transcription and transforming growth factor-beta activity through antagonism of cGMP-dependent protein kinase repression via upstream stimulatory factor 2. The Journal of biological chemistry.

[54] Putaala H, Soininen R, Kilpelainen P, Wartiovaara J, Tryggvason K (2001). The murine nephrin gene is specifically expressed in kidney, brain and pancreas: inactivation of the gene leads to massive proteinuria and neonatal death. Hum Mol Genet.

[55] Roselli S (2004). Early glomerular filtration defect and severe renal disease in podocin-deficient mice. Mol Cell Biol.

[56] Theilig F (2001). Cellular distribution and function of soluble guanylyl cyclase in rat kidney and liver. Journal of the American Society of Nephrology: JASN.

[57] Lewko B (2015). Dexamethasone-dependent modulation of cyclic GMP synthesis in podocytes. Molecular and cellular biochemistry.

[58] Wang S, Mitu GM, Hirschberg R (2008). Osmotic polyuria: an overlooked mechanism in diabetic nephropathy. Nephrology, dialysis, transplantation: official publication of the European Dialysis and Transplant Association - European Renal Association.

[59] Luippold G, Bedenik J, Voigt A, Grempler R (2016). Short- and Longterm Glycemic Control of Streptozotocin-Induced Diabetic Rats Using Different Insulin Preparations. PloS one.

[60] Ott IM (2012). Effects of stimulation of soluble guanylate cyclase on diabetic nephropathy in diabetic eNOS knockout mice on top of angiotensin II receptor blockade. PloS one.

[61] Schneider CA, Rasband WS, Eliceiri KW (2012). NIH Image to ImageJ: 25 years of image analysis. Nature methods.

