# Supplementary material for: Cinaciguat ameliorates glomerular damage by reducing ERK1/2 activity and TGF-ß expression in type-1 diabetic rats
Source: Sci Rep. 2017 Sep 11;7:11218. doi: 10.1038/s41598-017-10125-3 (PMC5593847; doi:10.1038/s41598-017-10125-3)
Supplement: Supplementary file 1 — Supplementary Figures [file 41598_2017_10125_MOESM1_ESM.pdf]

Supplementary Information

Cinaciguat ameliorates glomerular damage by reducing ERK1/2 activity and TGF-ß expression in type-1 diabetic rats

Szabina Czirok MD[a], Lilla Fang MD PhD[a], Tamás Radovits MD PhD[b], Gábor Szabó MD PhD[c], Gábor Szénási MSc PhD[a], László Rosivall MD DSc[a], Béla Merkely MD DSc[b], Gábor Kökény MD PhD[a*]

[a]Institute of Pathophysiology, Semmelweis University, Budapest, Hungary

[b]Heart and Vascular Center, Semmelweis University, Budapest, Hungary

[c]Department of Cardiac Surgery, University of Heidelberg, Heidelberg, Germany

Corresponding author:

Gábor Kökény, M.D, Ph.D.

Institute of Pathophysiology, Semmelweis University,

Nagyvárad tér 4., H-1089 Budapest, Hungary

Phone: +36-1-210-2930/56481; Fax: +36-1-210-0100;

E-mail: kokeny.gabor@med.semmelweis-univ.hu

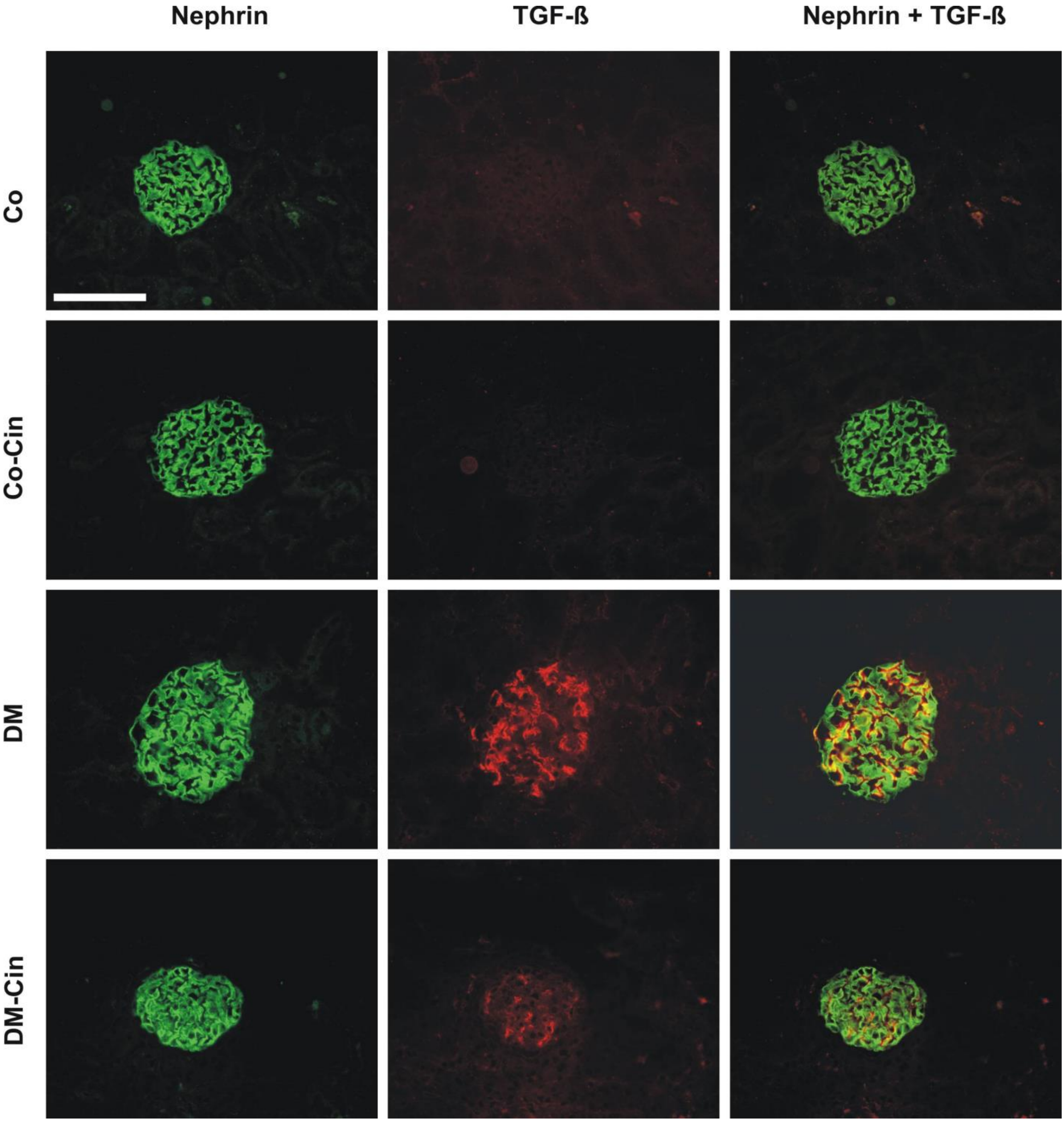

Supplementary Figure 1. Effect of diabetes and cinaciguat on glomerular TGF-β immunostaining. Representative photomicrographs of double immunostaining for TGF-β (red) and nephrin (green) depicted complete absence of glomerular TGF-β in both Co and Co-Cin kidneys, but a marked mesangial TGF-β immunoreactivity in DM kidneys, which was attenuated by cinaciguat treatment, as seen in DM-Cin glomeruli. Bar represents 50 µm (400x magnification).

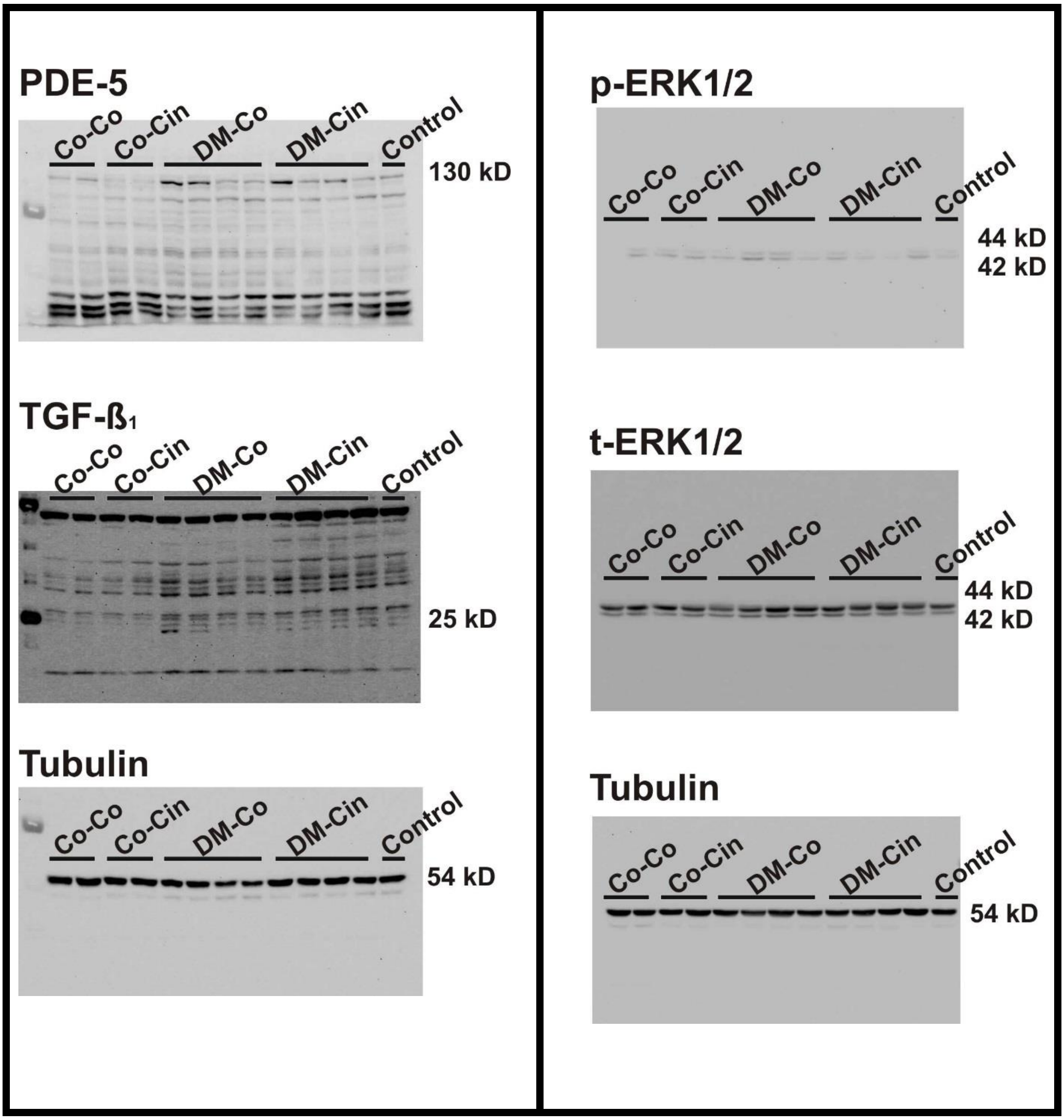

Supplementary Figure 2. Representative immunoblots of PDE-5, TGF-ß1 and p-ERK1/2 showing the specific bands.

The last Control band on every blot served to normalize signals between blots incubated with the same antibody, after normalized to the corresponding tubulin chemiluminescence signal. PDE-5: phosphodiesterase-5; TGF-$ß_1$: transforming growth factor-$ß_1$; ERK1/2: extracellular signal regulated kinase 1/2

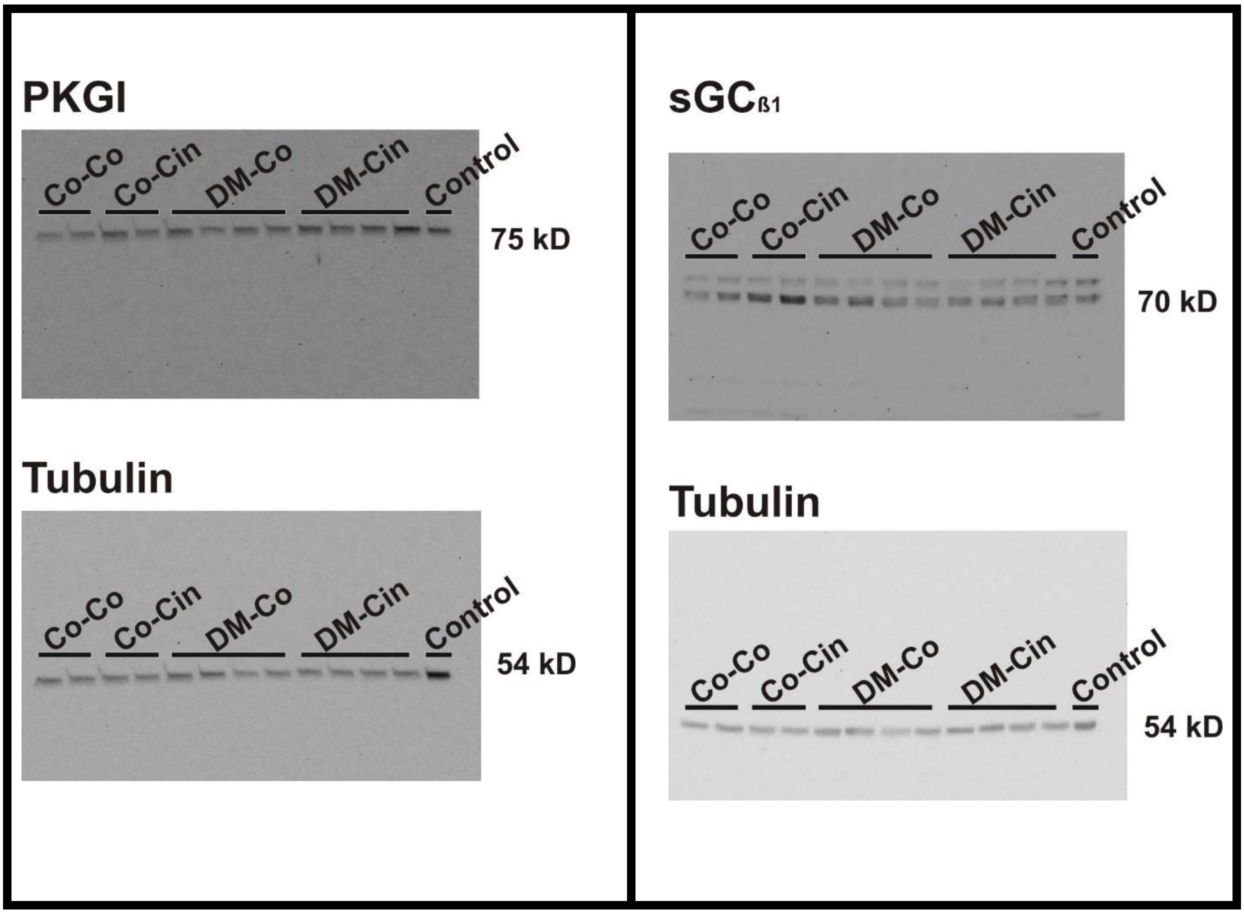

Supplementary Figure 3. Representative immunoblots of PKGI and sGC$_{ß1}$ showing the specific bands.

The last Control band on every blot served to normalize signals between blots incubated with the same antibody, after normalized to the corresponding tubulin chemiluminescence signal.

PKGI: prorein kinase G type I; sGC$_{ß1}$: soluble guanylyl cyclase ß subunit-1

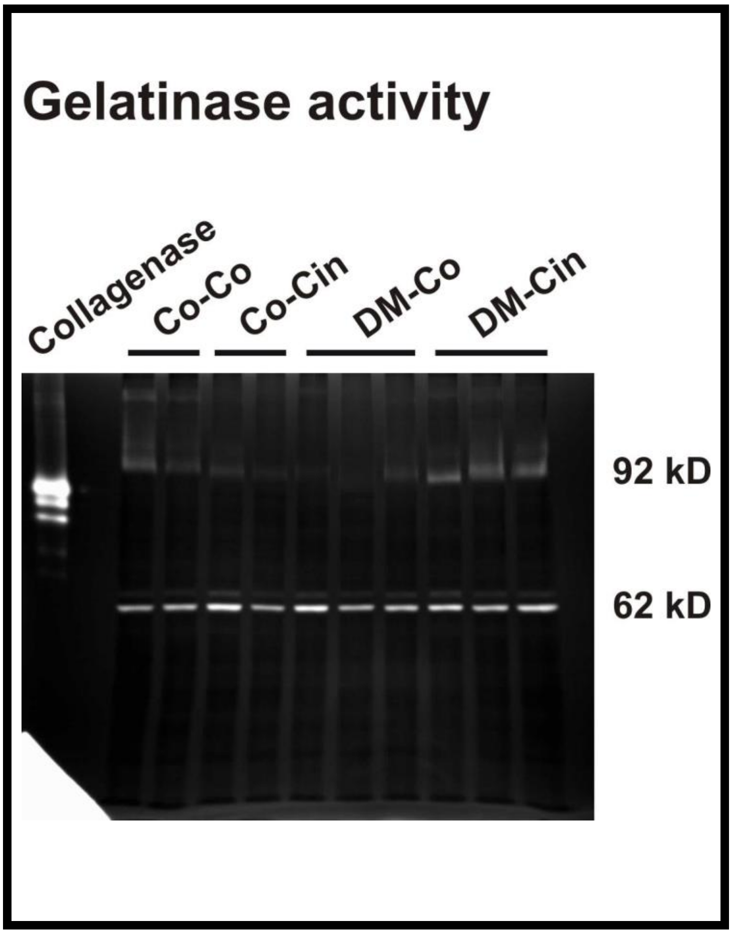

Supplementary Figure 4. Representative zymogram showing MMP-9 (92 kD) and MMP-2 (62 kD) gelatinase activity.

The first Co-Co sample was used on multiple zymograms to normalize gelatinase activity between gels. Collagenase served as positive control.
